# Practical Points in the Treatment of Hip-Disease

**Published:** 1894-04

**Authors:** 


PRACTICAL POINTS IN THE TREATMENT OF HIP-DISEASE.—Judson (*Med. Rec.; XLIV.*; No. 18). The same general rules or principles of treatment hold good in the earliest stage and in the most desperate and advanced stages.

Treatment should make sure of four things: (1) Preservation of the health throughout the treatment; (2) the arrest of motion in the joint in the acute stage; (3) the removal of the weight of the body from the joint in all stages; (4) provision for final symmetry and locomotor ability.

Under the fourth heading he says: The patient, while wearing the hip-splint may be induced by instruction and drill to give up the "false time" of his footsteps, which is the chief cause of the deformity and limp of hip-disease, and to return to the natural rhythm of human locomotion, in which equal time is given to the two feet. The result of walking habitually in this way is the abolition at once of a great deal of the appearance of lameness and in time the correction of deformity, because the adducted limb will pass, without conscious effort on the part of the patient, from adduction to abduction (with, at the same time, and for the same reason, a decrease in flexion), in order to place itself in that position in which it can best do its half of the work of progression, thrown upon it by the adoption, or rather the resumption, of natural rhythm or correct time in walking.

In reference to the question of traction, Judson says: The hip-splint is to be worn day and night. It makes traction on the limb and protects the joint from the traumatisms of walking. The latter is necessary in all stages of the disease, but there are long periods when traction is not necessary, when all that the patient requires is a perineal or ischiatic support to keep the affected limb from reaching the ground. The adhesive plasters and the rack and pinion and other machinery for maintaining traction may therefore be dispensed with during a large part of the treatment, and the joint be protected only by a simple ischiatic crutch which may, like any other crutch, be laid aside at night. [The Editor cannot refrain from urging the utmost care in deciding when traction can be safely omitted; and also when the splint can be laid aside at night. So long as the least reflex muscular spasm can be detected, the joint needs constant protection.]—*American Medico-Surgical Bulletin.*

